# Sharp temporal tuning in the bat auditory midbrain overcomes spectral-temporal trade-off imposed by cochlear mechanics

**DOI:** 10.1038/srep29129

**Published:** 2016-07-04

**Authors:** Silvio Macías, Julio C. Hechavarría, Manfred Kössl

**Affiliations:** 1 Institut für Zellbiologie und Neurowissenschaft, Goethe Universität Frankfurt am Main, Germany

## Abstract

In the cochlea of the mustached bat, cochlear resonance produces extremely sharp frequency tuning to the dominant frequency of the echolocation calls, around 61 kHz. Such high frequency resolution in the cochlea is accomplished at the expense of losing temporal resolution because of cochlear ringing, an effect that is observable not only in the cochlea but also in the cochlear nucleus. In the midbrain, the duration of sounds is thought to be analyzed by duration-tuned neurons, which are selective to both stimulus duration and frequency. We recorded from 57 DTNs in the auditory midbrain of the mustached bat to assess if a spectral-temporal trade-off is present. Such spectral-temporal trade-off is known to occur as sharp tuning in the frequency domain which results in poorer resolution in the time domain, and vice versa. We found that a specialized sub-population of midbrain DTNs tuned to the bat’s mechanical cochlear resonance frequency escape the cochlear spectral-temporal trade-off. We also show evidence that points towards an underlying neuronal inhibition that appears to be specific only at the resonance frequency.

Temporal information extracted from sounds by the auditory system is crucial for a wide range of behaviors including communication, prey detection, and predator avoidance. It is therefore not surprising that the representation and analysis of temporal parameters is a fundamental property of the auditory system across taxa. Among temporal parameters, sound duration is an important feature of many acoustic signals^1^,^2^. For example, stereotypy in syllable and/or phoneme duration is critical for the recognition of human speech^3^. In mammals, as in many other vertebrates, there are neurons tuned to sound duration, so called duration-tuned neurons (DTNs)^4^. Studies on anurans^5^ and echolocating bats^6^ have provided thorough associations between behaviorally relevant acoustic signals (i.e. communication and/or echolocation signals) that vary in duration, and their neural representations via the response of DTNs.

DTNs, which are also selective for stimulus frequency, can be viewed as spectro-termporal filters responsible for the analysis of both frequency and time at the single neuron level. There is evidence indicating that the frequency-tuning sharpness of neurons is affected by their duration-tuning characteristics^7^. However, only one study have addressed the interaction and trade-offs that occur between frequency and duration tuning in single neurons that are tuned to both of these parameters^8^.

It is known that in the case of resonant filters, a trade-off between spectral and duration representations occurs if sounds are sampled over long time windows, by which more cycles of the signal are added to increase the accuracy of frequency estimation. In the cochlea of the mustached bat, *Pteronotus parnellii*, the region that operates as a resonant filter is known as the “auditory fovea”, and in this region, frequency encoding resolution could be as sharp as a few hundred Hz^9^. At the frequency of cochlear resonance, the cochlear microphonic responses of *P. parnellii* show oscillations lasting several milliseconds following the stimulus offset. Such phenomenon is known as “cochlear ringing”, and cochlear ringing is thought to be responsible for afterdischarges lasting between 10–15 ms in some neurons from the cochlear nucleus that are sharply tuned to the cochlear resonance frequency^10^. In other words, in animals such as *P. parnellii,* representing frequency with high precision seems to be achieved in the cochlea at the expense of losing the ability of precisely encoding sound offsets, a feature that is inherited by central neurons. According to the models that explain the central implementation of duration tuning, precise sound offset encoding is instrumental for the formation of sharp duration tuning at the level of the midbrain^11^. Therefore, one could hypothesize that in animals such as *P. parnellii*, the sharpness of sound duration encoding shall be poor in neurons that are sharply tuned to frequency.

To test the aforementioned hypothesis, we recorded from neurons that were sharply tuned to frequency while at the same time being tuned to sound duration. Since these neurons first appear at the level of the midbrain, where duration tuning is computationally implemented^12^, we focused on this auditory station. This species was chosen because of its interesting flutter-detection echolocation and Doppler-shift compensation behavior. The dominant frequency of the outgoing echolocation calls for a stationary (e.g. roosting) mustached bat is called its resting frequency; however, flying mustached bats adjust the frequencies of their calls so that the returning (and Doppler-shifted) echoes always fall within the cochlea’s region of highest sensitivity and frequency resolution.

The results presented in this paper show that, contrary to what was expected, midbrain duration tuned neurons of the mustached bat could actually achieve sharp duration encoding accuracy while at the same time being sharply tuned to frequency. We also show indirect evidences from extracellular recordings that suggest the existence of a frequency-specicific inhibition operanting at the bats’ resonance frequency. Such inhibition could allow midbrain auditory neurons to overcome mechanical limitations imposed by cochlear mechanics to temporal auditory processing.

## Results

**Figure 1:**
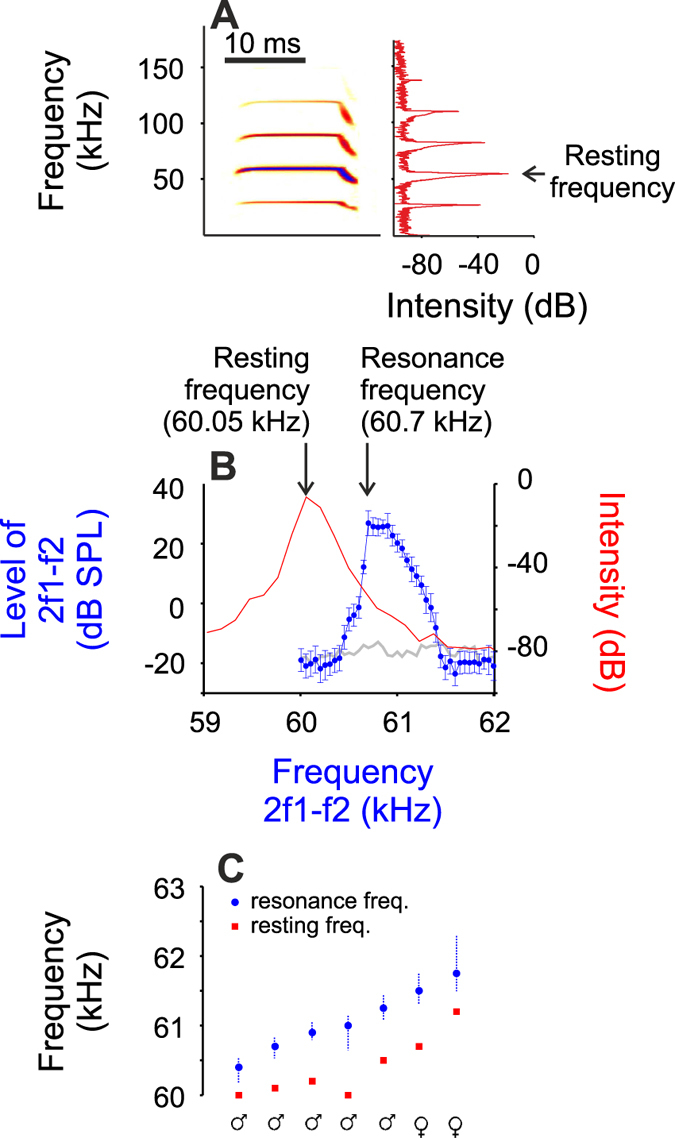
Resting and resonance frequencies. (**A**) Sonogram (time vs frequency representation, left) and power spectrum (right) of a call emitted by a bat resting in a cage. (**B**) Averaged DPOAE audiogram (in blue) calculated in the same bat measured five times. Grey line indicates the average noise level. A close up (between 59 and 63 kHz) of the echolocation call represented in A is represented in red. Values of the resonance and the resting frequency for that individual bat are shown. (**D**) Values and ranges of the resonance frequency (blue circles and blue dashed lines) and value of the resting frequency (red squares) measured from all bats. Male and females are ordered according to increasing resonance frequency.

In each individual bat, prior to neurophysiological recordings, we measured the peak frequency of the constant frequency component of the second harmonic (CF2) while the animals were resting (non-flying) in a cage. This frequency is referred to as the resting frequency^13^, and it varied between 60 and 61.2 kHz among individual bats (mean = 60.38 ± 0.44 kHz). Shortly after measuring the resting frequency, the cochlear resonance frequency was measured using distortion product otoacoustic emissions (DPOAEs). An average DPOAE audiogram (blue trace) calculated from five trials in one female bat is shown in Fig. 1B. The cochlear resonance frequency was defined as the 2f2-f1 frequency at the peak DPOAE level. In addition, a resonance range was defined, by interpolation, as the range that encompassed frequencies in which the DPOAE audiogram was above 75% of the peak DPOAE level. In all bats, the resonance frequencies were higher than the resting frequencies (Fig. 1C), as it would be expected from previous studies^10^.

**Figure 2:**
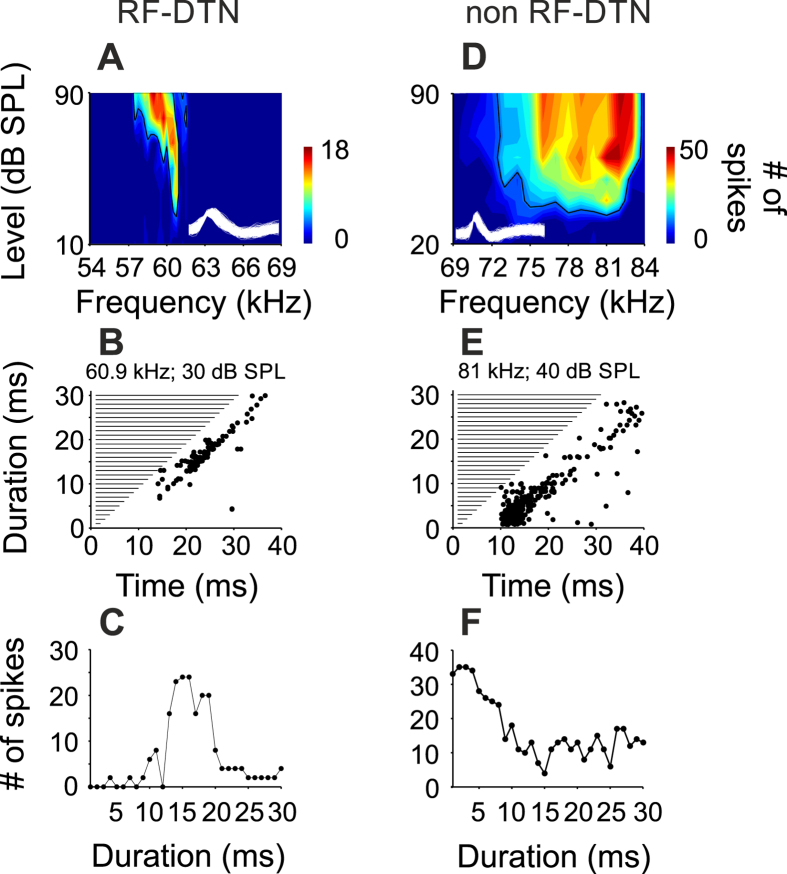
Frequency and duration tuning of neurons in the IC of the mustached bat. (**A–C**) RF-DTN showing a band-pass characteristic. (**D–F**) Non RF-DTN with short-pass duration tuning. For both neurons is shown the FRA (top), the duration response dot-raster display (middle) and the duration-response function (bottom). Two milliseconds of the action potential waveforms (y-axis in arbitrary units) obtained after spike sorting are shown inside the FRAs.

**Figure 3:**
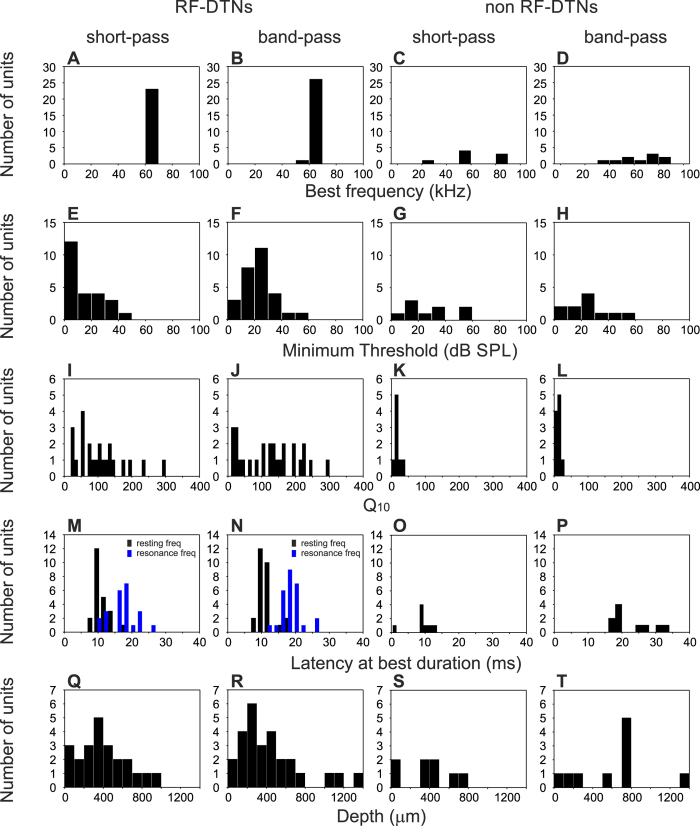
Distribution of physiological properties of DTNs in the IC of the mustached bat.

**Figure 4:**
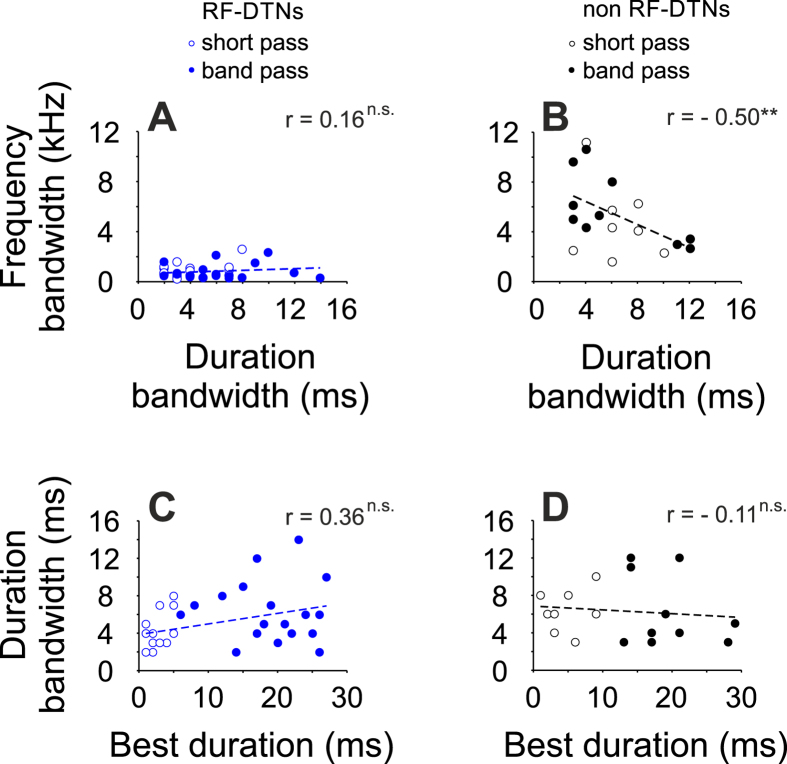
(**A,B**) Relation of spectral and temporal tuning. RF-DTNs (n = 37) tuned to sound duration at the resonance frequency. There was no significant correlation between frequency bandwidth and duration bandwidth, suggesting that there is no trade-off in spectral-temporal resolution. Non RF-DTNs (n = 18) tuned to sound duration at the best frequency. There is a significant correlation between frequency and duration bandwidth. (**C,D**) Relation of best duration to duration bandwidths.

Reported here are data from 57 DTNs in the IC of seven bats. These neurons were part of a population of 202 neurons, in which 145 showed no duration selectivity. Units responsive to pure tones were located at depths between 10 and 1200 μm from the surface of the IC. The best frequency (BF) of units ranged from 27 to 91 kHz. IC units were divided into two groups depending on their BFs. In each DTN, we measured the frequency bandwidth (bandwidth of the threshold curve at 10 dB above the response threshold) and the duration bandwidth (measured by interpolation as the difference between the shortest and longest stimulus durations, where the response function obtained at +10 dB relative to threshold dropped to ≤50% of the peak response). The first group included 41 units tuned with BFs falling within the resonance frequency range, from here on referred to as RF-DTNs. Figure 2A–C shows an example of a RF-DTN with a BF of 60.9 kHz (frequency bandwidth = 0.75 kHz) tuned to a best duration of 15 ms with a duration bandwidth of 7 ms. The second group, 16 units, had BFs outside of the resonance frequency range (non RF-DTNs). An example is given in Fig. 2D–F. This neuron had a broad frequency response area (frequency bandwidth = 9 kHz) with a BF of 81 kHz. This neuron showed a best duration = 3 ms, with a duration bandwidth of 8 ms (between 1 and 9 ms). This distinction was based solely on whether the best excitatory frequency fell within the bat’s cochlear resonance frequency (RF) band, although RF-DTNs were more sensitive, with most of them showing minimum thresholds around 0 dB SPL and were most sharply tuned with Q10 values (value characterizing the sharpness of the FTC) between 10 and 298. On the other hand, non RF-units showed a sparser minimum threshold distribution and lower Q10 values. The distribution of basic responses of sampled neurons are shown in Fig. 3. Overall, RF-DTNs displayed duration tuning bandwidths ranging from 2 to 14 ms and there was no evidence of a trade-off between spectral and temporal resolution. For example, there was no negative correlation between the bandwidth of duration and frequency tuning (r = 0.016; p = 0.185; Fig. 4A). A different result was found for the population of non RF-DTNs (Fig. 4B). Here, we found a significant negative correlation between bandwidth of frequency and duration tuning (r = −0.50; p < 0.001). The latter indicates that non RF-DTNs with narrower duration tuning show wider frequency tuning, and vice versa and the spectral-temporal relation for the two neuronal subgroups does not depend on whether the units are short-pass or band-pass duration-tuned. We examined how the best duration relates to temporal bandwidth for the two subpopulations of DTNs units (Fig. 4C,D). For both types of units (that is RF-DTNs and non-RF DTNs), there was no significant correlation between best duration and duration bandwidth.

**Figure 5:**
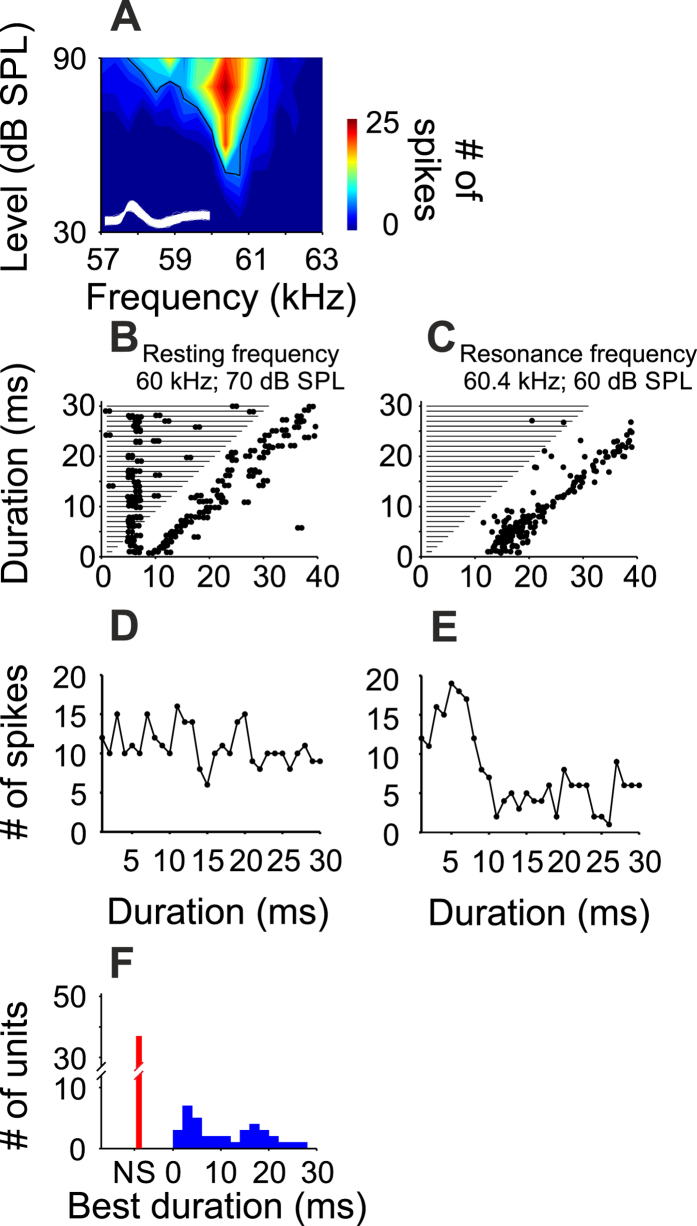
Tuning curve and duration tuning properties of a RF-DTN. (**A**) FRA. Two milliseconds of the action potential waveforms (y-axis in arbitrary units) obtained after spike sorting are shown inside the FRA. (**B,C**) Duration response dot-raster display in response to the resting and the resonance frequency. (**D,E**) Duration-tuning curves measured at the resting and resonance frequencies. (**F**) Distribution of best durations measured in 37 RF-DTNs in response to the resting frequency (red bars) and resonance frequency (blue bars). NS: no duration selectivity.

The influence of the stimulus frequency on duration tuning was examined in 37 of the 41 RF-DTNs. For each RF-DTN, we tested the duration selectivity in response to the unit’s best frequency, which was in the range of the bat’s resonance frequency, and in response to the bat’s resting frequency. An example neuron is shown in Fig. 5. RF-DTNs were not duration tuned when the resting frequency was used as a stimulus (Fig. 5B,D) but they had a clear duration selectivity when tested using the resonance frequency (Fig. 5C,E). The distribution of best durations (stimulus duration eliciting the maximum response) of the population of RF-DTNs is shown in Fig. 5F. Best durations obtained at the resonance frequency followed a bimodal distribution, with a peak at 4 ms and a second peak at 17 ms (Fig. 5F, blue bars). There was no evidence of duration selectivity when stimulated with the resting frequency (Fig. 5F, red bars).

**Figure 6:**
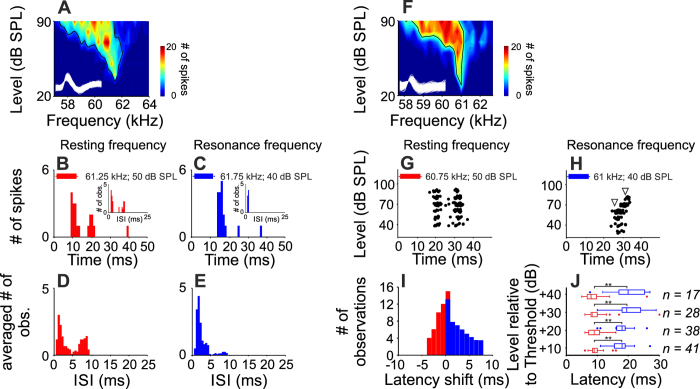
Temporal characteristics of the response of DTNs. (**A–C**) FRA (**A**) and response pattern of a DTN in response to the resting (**B**) and the resting frequency (**C**). (**B**) PSTH in response to a 10 ms tone burst set at 10 dB above threshold for the resting frequency. Inset: Inter-spike interval histogram. (**C**) PSTH in response to a 10 ms tone burst set at 10 dB above threshold for the resonance frequency. Inset: Inter-spike interval histogram. (**D,E**) Average inter-spike interval histograms calculated for the response obtained at 10 dB above threshold for the resting frequency and the resonance frequency in 41 RF-DTNs. Histograms were constructed with bin widths of 1 ms. (**F–H**) FRA and dot raster histogram representing the timing of a spike relative to the stimulus onset with increasing sound level, obtained at the resting (**G**) and the resonance frequency (**H**). Two milliseconds of the action potential waveforms (y-axis in arbitrary units) obtained after spike sorting are shown inside the FRAs. (**I**) Histograms of the maximum latency shift observed in each neuron with increasing sound level in 41 units at the resting frequency (red bars) and the resonance frequency (blue bars). (**J**) Comparison between the latency of the response to the resting frequency and to the resonance frequency at 10, 20, 30 and 40 dB above threshold in all sampled RF-DTNs. Asterisks represent significant differences (paired t-tests,p < 0.001). The number of neurons included in the comparison for each level are indicated.

We studied the temporal characteristics of the response of RF-units to the constant frequency component of bats emissions broadcasted in the resting state (the resting frequency) and to the cochlear resonance frequency measured with DPOAEs (the resonance frequency). Resonance frequency is particularly important for bats when processing echoes generated during flight, and resting frequency is important for processing the frequency of pulses and echoes generated when stationary. Within the excitatory tuning area, we found systematic frequency dependent changes in the temporal response characteristics. All RF-DTNs showed an on-off response at the bats’ resting frequency, whereas at the resonance frequency the neurons showed a phasic-like response. An example unit whose response illustrates these frequency-dependent response pattern is shown in Fig. 6A–C. Frequency-dependent response patterns were also apparent in the averaged interspike interval calculated in all RF-DTNs for both the animal specific biosonar resting frequency and the cochlear resonance frequency (Fig. 6D,E).

The effect of sound level on response latency was also frequency-dependent. For example, at the resting frequency, the unit depicted in Fig. 6F–H responded with a latency of 19 ms that remained constant at sound levels from 40–80 dB SPL. The latency of this unit decreased by 1 ms at 90 dB SPL (Fig. 6G). At the cochlear resonance frequency, the response latency of the same unit decreased from 27 to 25 ms between 30 and 60 dB SPL, and a further increase of the amplitude evoked a latency increase that shifted to 31 ms at 80 dB SPL, a phenomenon defined as paradoxical latency shift, PLS (Fig. 6H)^14^. When stimulated with the resting frequency, in 28 of 41 units (~68%) the response latency systematically decreased with increasing sound level. In these units the latency was reduced by 2.0 (±1.2) ms per 10 dB increment. This latency decrement was most evident within the range of up to 30 dB above the response threshold. The remaining 13 units showed a constant response latency independent of rising sound level and within this subset of neurons, response latency did not vary by more than 1 ms from one stimulus amplitude to the next, as observed in the histogram of the maximum latency shift observed in each neuron (Fig. 6I, red bars).

In contrast to the latency behavior observed in response to the resting frequency, when stimulated with the cochlear resonance frequency, in 30 of the 41 studied units, the increment of stimulus amplitude evoked an increase in the response latency. In all 30 units, there was a progressive shift of the response latency and the PLS magnitude at the resonance frequency varied from 1 to 7 ms (mean = 3.89 ± 1.88 ms, Fig. 6I, blue bars). In addition, at each tested sound level, latency measured at the resonance frequency was significantly longer than that measured at the resting frequency (paired t-test for each level, p < 0.001, power of test = 0.9, Fig. 6J).

## Discussion

While fine analysis in the frequency domain is important for auditory processing, a good resolution in the time domain is also essential. In echolocating bats, neuronal filters tuned to both sound duration and frequency could help to maximize the extraction of acoustic information from reflected echoes and/or trigger fixed action patterns associated with foraging^15^. According to our results, a subpopulation of DTNs (RF-DTNs) in the IC of the mustached bat are able to encode sound properties with high resolution in both the frequency and the time domain and the absence of a spectral-temporal trade-off for duration selectivity offers an excellent example of this ability. This contrasts with the spectral-temporal trade-off observed in non RF-units of the mustached bat that are also duration-tuned (non RF-DTNs), and with the properties of DTNs found in the IC of the big brown bat, *Eptesicus fuscus*^8^. Non RF-DTNs in the IC of the mustached bat and collicular DTNs in *Eptesicus* with wider frequency bandwidth display narrower duration bandwidth and viceversa. In the mustached bat IC, neurons tuned to the resonance frequency inherit an extremely sharp frequency tuning from mechanical adaptations in the periphery. According to our results, such mechanical adaptations found in the periphery do not impose limitations to the duration encoding resolution of RF-DTNs, since their duration tuning curves span bandwidths that are no-different from those found in non RF-DTNs (see Fig. 2G,H). Neurons tuned to the resonance frequency maintain high temporal precision when other features are analyzed. For example, facilitated delay tuning in these units is as sharp as in non RF-neurons^16^. For this to occur, the temporal precision of the best frequency response in RF-neurons has to be as great as for the best frequency response in non RF-neurons.

In each studied specimen, the biosonar resting frequency and the cochlear resonance frequency differed by only a few hundred Hertz, yet this small frequency difference evokes large changes in the neuronal responses of IC neurons, in terms of duration tuning, temporal response pattern, and latency-amplitude functions. This indicates the existance of underlying synaptic mechanisms operating only at the resonance frequency, which may be responsible for the absence of a spectral-temporal trade-off in RF-DTNs. Our results suggest the existence of a frequency-specific inhibition that differentially controls the shape of neuronal responses to different frequencies. The presence of duration tuning and PLS only in response to the resonance frequency could be considered as indicators of this inhibition, since inhibition is known to participate in the formation of both of these neuronal traits (see below^12^,^17^). However, we should be aware that there is no evidence that the inhibition involved in the occurrence of PLS, which has high threshold and could be from a different brainstem source with different response properties, is related to the inhibition in duration tuning.

We observed that RF-DTNs were duration tuned at the resonance frequency, but not at the resting frequency. Within the IC, duration selectivity is putatively created through the convergence and temporal interaction of excitatory and inhibitory synaptic inputs that are offset in time^4^,^12^,^18^,^19^,^20^,^21^,^22^,^23^,^24^,^25^. Two different models could explain duration selectivity: coincidence^12^,^19^ and anti-coincidence mechanisms^20^. Basically the same components are involved in both models: a short-latency inhibitory input that persists for the duration of the stimulus; a delayed excitation triggered at stimulus onset; and an excitatory rebound from inhibition. In the coincidence model, a response appears only when the rebound from the inhibitory component coincides and sums with the delayed excitation. In the anti-coincidence model, early inhibition does not contribute to excitation (the inhibitory rebound is not present). At short stimulus durations, inhibition ceases before the arrival of the excitatory input, and the neuron responds maximally. Most, if not all, DTNs studied in the IC of bats may derive their selectivity through one of these mechanisms, which are not mutually exclusive^4^,^12^,^18^,^19^,^20^,^21^,^24^,^25^,^26^,^27^. In both models, inhibition is an important component. When inhibition acting on a DTN is diminished or blocked, a cells duration selectivity is severely reduced (i.e. broadened) or abolished^12^,^18^,^20^,^28^,^29^,^30^. Mechanical cancellation in the cochlea, which results during on-off responses, could be responsible for the absence of duration selectivity at the resting frequency. This mechanical suppression may prevent neurons at subcollicular levels from developing a sustained inhibition that is necessary for the implementation of duration tuning in collicular neurons. The opposite occurrs at the resonance frequency, where neurons from the cochlear nucleus display a tonic primary-like response. This response, that lasts throughout the stimulus duration, and even longer in the case of ringing, could provide the necessary inhibition for the occurrence of duration tuning in the IC.

RF-DTNs showed different response properties depending on stimulus frequency. At the resting frequency, neurons responded with an on-off pattern. This on-off phenomena, also observed in the cochlear nucleus^10^, could be produced in the cochlea by mechanical cancellations restricted to a narrow frequency range. In the cochlea of the mustached bat, the tectorial membrane is locally enlarged in the second half turn where the 61 kHz range is represented^31^. In particular, at a basilar membrane position slightly basal to the representation of the resonance frequency, the attachment of the tectorial membrane to the spiral limbus is largely reduced and could be instrumental as a resonator that enhances senstitivity and sharp tuning at the resonance frequency^32^,^33^. At slightly lower frequencies (i.e. resting frequency) resonance energy cancels incoming oscillations and may produce on-off responses^34^. Shortly after the onset of the stimulus, cancellations would suppress tonic activity, whereas following the end of the stimulus, release of suppression or resonant activity would produce an offset response^35^. The latter pattern could lead to the neuronal temporal response observed in the IC and the cochlear nucleus at the resting frequency. At the resonance frequency, the temporal response pattern of collicular RF-DTNs is phasic in contrast to the tonic and long-lasting response of neurons in the cochlear nucleus tuned to the resonance frequency. This change in the response pattern is due to GABA-mediated lagging inhibition. For example, a previous study showed that when GABAergic inhibition is blocked, IC units fire 20 or more spikes in response 20-ms stimuli, while in the baseline situation they fire only l to2 spikes for each stimulus regardless of its duration^36^. These response features suggest that there is a temporal interaction between a strong tonic excitatory drive and strong inhibition, where the full strength of the inhibition is imposed with a latency slightly longer than the excitation. Our results complement this previous study by showing that such inhbitory effects are frequency-specific, as they occur only at the cochlear resonance frequency.

The occurrence of PLS only at the resonance frequency could be considered as further evidence of the underlying frequency-specific inhibition. PLSs appear to be created by high-threshold inhibition^17^,^37^,^38^,^39^,^40^. Intracellular recordings from IC neurons exhibiting PLS revealed that a cell’s response latency is prolonged at increasing sound levels due to the occurrence of a high-threshold inhibitory post-synaptic potential (ipsp) that suppresses the first portion of the responses and the response latency is therefore shorter^41^ (at lower sound levels the ipsp does not influence neuronal firing). Iontophoretic injection of bicuculline, an antagonist of GABAa receptors, in IC neurons displaying PLS abolishes it^17^,^37^. The functional role of PLS in RF-DTNs of the mustached bats IC remains unclear. PLS have been suggested as the neural mechanism driving the echo-delay selectivity in FM bats^37^,^38^,^42^. Whatever the function, PLS are a clear indication of the presence of an underlying inhibition that, in the case of the mustached bat, occurs only in response to the resonance frequency. Further evidence supporting the existence of a frequency specific inhibition is the fact that when neurons tuned to the 61 kHz frequency range are tested for delay tuning, most of them show inhibitory delay tuning curves^16^,^43^. We do have to emphasize that, although the evidence in favor of a frequency specific inhibition that shapes neuronal responses to the resonance frequency appears to be convincing, this inhibition needs to be studied in more detail, e. g. with intracellular recordings and/or by combining extracellular recordings and pharmacological experiments.

Data from non RF-DTNs in the IC of the mustached bat confirms the generality of the spectro-temporal resolution trade-off first reported by Morrison *et al*. (2014) for DTNs in *E. fuscus*, a bat that has neither an auditory fovea nor Doppler-shift compensation behavior^8^. On the other hand, the lack of a time-frequency resolution trade-off in RF-DTNs points to specializations within this neural sub-population that is likely to be highly relevant to the flutter detection and Doppler-shift compensation behavior of *P. parnellii*. It would be very interesting to know if the findings in *P. parnellii* -one of a handful of new world bats with an auditory fovea and Doppler-shift compensation- also hold true when tested in one of the many species of old world *Rhinolophus* or *Hipposideros* bats that have independently evolved an auditory fovea, flutter detection echolocation, and Doppler-shift compensation behavior.

The behavioral role of DTNs is not completely understood. One hypothesis is that, in bats, neuronal tuning for stimulus duration is a mechanism that operates during the processing of echolocation calls and/or echoes. The best duration and range of temporal selectivity of DTNs in bats closely mirrors the range of echolocation call durations^20^,^21^,^22^,^44^. It has been also suggested that duration tuning might play a role in echo recognition, since neuronal frequency tuning sharpness appears to improve when studied at the neurons’ best durations^7^,^45^. Also, DTNs might play a role in the analysis of sounds that are not self generated, such as communication sounds, which occupy a large portion of the mustached bat vocal repertoire^46^. It is, of course, possible that duration tuning could be an expression of neural mechanisms that analyze some other parameters besides sound duration, such as sweep rate and echo delay sensitivity. In these other selectivity forms inhibition is also a fundamental element^47^,^48^,^49^,^50^,^51^,^52^.

## Methods

### Animals

The study included seven adult *Pteronotus parnellii* (five males and two females). The animals were captured at the entrance of their diurnal refuge (a cave located 30 km southeast of Havana, Cuba) during their evening exodus and kept in captivity in a room with temperature, humidity and photoperiod conditions similar to those of the bats natural environment. The use of animal subjects and experimental procedures in this study were authorized by the Centre for the Inspection and Control of the environment, Ministry of Science, Technology and Environment, Cuba. All experiments were in accordance with the Declaration of Helsinki and also with German federal regulations. All the experimental protocols were approved by the Goethe University Animal Care and Use Committee.

### Acoustic measurenments

The resting frequency was recorded with a 1/4” Brüel and Kjaer 4135 microphone connected to an Avisoft Bioacoustics mobile recording interface (UltraSoundGate 381 system: 116Hm) and measured usingAvisoft 382 SASlab Pro software. DPOAEs were recorded in awake bats in a sound-attenuated chamber. To ensure that bats were not able to move during recording, their heads were fixed with a custom-made mouth holder. To measure DPOAE, an acoustic coupler was placed in the outer ear canal at a distance of about 0.3–1.0 mm from the tympanum under visual control (Zeiss OPMI 1-FR binocular, Carl Zeiss AG, Jena, Germany). The coupler consisted of three acoustic channels that converged at the coupler’s tip. Two of the coupler channels were connected to reversely driven condenser microphones used as loudspeakers (1/2“, MTG MK202, Microtech Gefell GmbH, Gefell, Germany) and the third channel contained a sensitive microphone (1/4“, B&K 4939, Brüel & Kjær, Nærum, Denmark) for recording DPOAE. A soundcard was used to generate the two pure tone stimuli and to record DPOAEs (RME fireface UC, RME Audio AG, Haimhausen, Germany; sampling rate: 192 kHz). Data acquisition and data analysis programs were written in MATLAB (MATLAB 2007b, MathWorks Inc.). The sound system was calibrated *in situ* using white noise. DPOAEs (2f1-f2) were recorded by varying the stimulus frequency f2 between 60 and 63 kHz, while maintaining an f2/f1 ratio of 1.001 and constant stimulus levels (L1 = 40 dB SPL, L2 = 30 dB SPL). This method yielded DPOAE audiograms (plots of DPOAE amplitude versus frequency), which were measured in each bat 4 or 5 times. Due to extremely sharp mechanical tuning in the mustached bats cochlea, this low f2/f1 ratio is suited to elicit the largest DPOAE levels^13^,^53^. From each DPOAE audiogram, the resonance frequency was defined as the DPOAE frequency at the maximum DPOAE amplitude.

### Neurophysiological recordings

Bats were prepared for surgery by anesthetizing them with a combination of ketamine and pentobarbital (15 mg/kg Ketavet® and 15 mg/kg Pentobarbital, Sigma) via a subcutaneous injection in the neck. A longitudinal midline incision was made through the skin overlying the skull, and the underlying temporal musculature was reflected from the incision along the midline. Wound surfaces were treated with a lidocaine solution applied topically. A custom-made metal rod was then glued to the skull using dental cement. We let the animals rest for 24 h before beginning electrophysiological recordings.

During neurophysiological recordings, awake bats were placed in a body mold made of plastic foam. Their head was tightly held by the rod fixed in a metal holder. Using skull and brain-surface landmarks, a small hole (of ≤~1 mm diameter) was made over the IC with a scalpel blade. A microelectrode (see following text) was then inserted through the hole in the skull. The hole was covered with saline solution during the experiment, and care was taken to prevent desiccation. The experiments were conducted inside a soundproof room (temperature: 27–32 °C) and lasted less than six hours. After each recording session, the exposed skull was covered with sterile bone wax, and the animal was returned to its individual cage. Bats could be studied for several consecutive days.

Carbon-fiber electrodes (Carbostar-1, Kation Scientific, 0.4–0.8 MΩ) were inserted perpendicular to the collicular surface. The depth of the electrode penetration was controlled using an externally controlled piezo manipulator (PM 10/1, Science Products GmbH, Hofheim, Germany). The electrical signal from the electrode was amplified (Differential amplifier EX-1, Dagan Corp.) and band-pass filtered between 200 Hz and 5 kHz. The neuronal signal was digitized by an A/D Microstar DAP 840 board (sampling rate 33 kHz), processed by a custom-written scripts, and stored for further analysis.

Acoustic stimuli were generated by the D/A board (DAP 840) at a sampling frequency of 278 kHz), attenuated (PA5, Tucker Davis Technologies), amplified (Avisoft Bioacoustics, portable ultrasonic amplifier), and delivered from a calibrated speaker (ScanSpeak Revelator R2904/7000, Avisoft Bioacoustics, Berlin, Germany). The speaker was placed at a distance of 10 cm from the contralateral ear. The speaker response was flat (±5 dB) at a frequency range between 10 and 100 kHz, and the amplitude of the presented pure tone stimuli (rise/fall time, 0.2 ms; repetition period, 250 ms) was adjusted in real time in accordance with the calibration frequency–response curve of the speaker. Auditory stimuli were controlled by custom-made software.

The calibration curve was obtained with a Brüel and Kjaer sound recording system (Microphone 4135, Microphone Preamplifier 2670) connected to a conditioning microphone amplifier (Nexus 2690). Briefly, the frequency–response curve of the stimulation system was determined by recording a band-passed noise (1–130 kHz) delivered through the stimulation speaker. Following this a 50 kHz pure tone was generated, and its amplitude was measured in an oscilloscope and compared with the amplitude of a 94 dB SPL-1 kHz tone generated by a calibrator (Brüel and Kjaer, 4231). The relative amplitude values (in dB) for frequencies between 1 and 130 kHz were transformed into sound pressure level values (in dB SPL) using the 50 kHz tone as a reference.

For each neuron the frequency response areas (FRA) were obtained using automatic stimulation. Neuronal responses to a 10 ms tone (0.2 ms rise-fall time) of randomly presented combinations of frequencies (10–125 kHz, 5–10 kHz steps) and intensities (0–90 dB SPL, 10 dB steps) were recorded in time windows of 160 ms. For neurons showing responses around the 61 kHz, a more detailed FRA (around the excitatory area with frequency steps of 50–250 Hz) was obtained. The limit of the FRA (threshold curve) was determined by linear interpolation (contourc function, MATLAB R2007a) as 25% of the maximum spike count obtained during the frequency-tuning protocol. The minimum threshold (MT) was defined as the lowest amplitude required for reaching 25% of the maximum spike count whereas the best frequency (BF) was calculated as the frequency at the response threshold. The sharpness of the frequency response areas was determined as the frequency bandwidth of the threshold curve at 10 dB above the response threshold. In RF-DTNs, we tested the influence of stimulus duration on individual bats’ resting and resonance frequencies. In neurons responding outside the 61 kHz range, we tested the effect of stimulus duration using the unit’s best frequency. Duration selectivity was assessed with tones at 10 dB above the MT for each frequency and durations between 1 and 30 ms (1 ms steps). To obtain both, the FRA and the duration tuning curve, each stimulus was presented 20 times.

Responses of each unit at resting resonance frequencies were analyzed offline and spikes obtained in response to all different stimulus combinations were sorted using the first three principle components of the spike waveforms^54^. This recorded multiunit activity was amplitude filtered so that only those spikes whose amplitude was at least three standard deviations above the recording baseline were used for subsequent analysis. To separate spikes originating from different neurons, an automatic clustering algorithm “KlustaKwik”^55^ was used, in which we inputted the first three principle components of each spike. This method has been successfully used to discriminate single-unit from multiunit activity in other studies^43^,^56^,^57^. Response latency was measured at the onset slope of the neuronal response, corresponding to the time at which 50% of the maximum post-stimulus time histogram (PSTH) was reached. PSTHs were constructed with a bin-size of 1 ms. Dot raster histograms (i.e. spike-times relative to the stimulus onset vs. stimulus amplitude) representing temporal response patterns were calculated for each neuronal response. Inter-spike interval histograms and latency-amplitude functions (i.e. response latency as a function of the stimulus amplitude) were also calculated.

## Additional Information

**How to cite this article**: Macías, S. *et al*. Sharp temporal tuning in the bat auditory midbrain overcomes spectral-temporal trade-off imposed by cochlear mechanics. *Sci. Rep.* **6**, 29129; doi: 10.1038/srep29129 (2016).
